# Antipsychotic medication and tobacco use among outpatients with schizophrenia: a cross-sectional study

**DOI:** 10.1186/1744-859X-13-7

**Published:** 2014-03-19

**Authors:** Hiranya Wijesundera, Raveen Hanwella, Varuni A de Silva

**Affiliations:** 1 University Psychiatry Unit, National Hospital of Sri Lanka, Colombo 08, Sri Lanka; 2 Department of Psychological Medicine, Faculty of Medicine, University of Colombo, Kynsey Road, Colombo 08, Sri Lanka

## Abstract

**Background:**

Many studies have shown that the prevalence of smoking in schizophrenia is higher than in the general population. Biological, psychological and social factors influence smoking in patients with schizophrenia.

**Methods:**

The study was carried out in psychiatry outpatient clinics in a tertiary care hospital in Sri Lanka. Every third patient was selected using systematic sampling from patients diagnosed with schizophrenia according to ICD-10 clinical criteria. Smoking behaviours were assessed using self-reports. Severity of illness was assessed using Brief Psychiatric Rating Scale (BPRS). Fagerstrom Test for Nicotine Dependence assessed level of dependence. Readiness to Change Questionnaire assessed motivation to change smoking behaviour.

**Results:**

The sample consisted of 306 patients with schizophrenia. Mean age was 38.93 years (SD 10.98). There were 148 males (48.4%). Mean duration of illness was 12.63 years (SD 8.38). Current medication was oral atypical antipsychotics 103, clozapine 136, oral typicals 29 and depot typicals 38. Prevalence of tobacco use among males was 30.41% (95% CI 22.91 to 37.90) and among females 1.90% (95% CI -0.25 to 4.05). Prevalence of current smoking among males was 20.27% (95% CI 13.72 to 26.82). None of the females smoked. Prevalence of smokeless tobacco use among males was 10.14 (95% CI 5.22 to 15.05) and among females 1.90 (95% CI -0.03 to 4.05). When patients treated with clozapine were excluded from the analysis, prevalence of tobacco use was 41.6% among males and 3.2% among females and prevalence of smoking was 29.9% among males. Prevalence of tobacco use was lowest in patients treated with clozapine 18.31 (95% CI 9.09 to 27.53) and highest in those treated with depot antipsychotics 47.83 (95% CI 25.74 to 69.91).

**Conclusions:**

Prevalence of smoking was less than in many countries. This is influenced by prevalence in the general population and low affordability. Risk of tobacco use was significantly less among patients treated with clozapine.

## Background

A recent report by the Royal College of Physicians and the Royal College of Psychiatrists in the UK highlights the problem of smoking among the mentally ill [1]. Many studies have shown that the prevalence of smoking in schizophrenia is higher than in the general population [2-6]. Smoking has health implications for patients with schizophrenia because it increases the risk of cardiovascular disease and malignancies and causes overall poor physical health and premature death. Smoking in patients with mental illness is also important because it increases the metabolism of medication through induction of the liver cytochrome P450 enzymes. It is necessary to identify factors which increase risk of smoking in mental illness, in order to implement preventive measures in this population [7].

A meta-analysis of prevalence of smoking in patients with schizophrenia, in different cultures, shows strong association between schizophrenia and current smoking (weighted average odds ratio, OR = 5.9) [8]. The prevalence of smoking in different countries ranges from 14% to 88%, with majority reporting prevalence between 40% and 70%.

Biological, psychological and social factors influence smoking in patients with schizophrenia. There is evidence of increased genetic vulnerability [9]. The dorsal anterior cingulate (dACC) functional circuits are key paths for increased risk of smoking co-morbidity in schizophrenia [10].

It has been suggested that nicotine may be used as self-medication for psychotic symptoms of schizophrenia [11]. However, smokers have higher Brief Psychiatric Rating Scale (BPRS) scores and experience more hospitalizations [12]. Nicotine may compensate for cognitive deficits in schizophrenia by improving cognitive functions such as attention and vigilance, spatial organization and visuospatial memory [13-17]. Nicotine increases midbrain dopaminergic activity, which may reduce negative symptoms in schizophrenia and activate a reward system [18,19]. Smoking may also be perceived as alleviating anxiety and mood symptoms by those who are dependent because it relieves unpleasant withdrawal symptoms [20]. A study of motivators for smoking in schizophrenia found that sedation and anxiety reduction, enhancement of pleasurable feelings and relaxation, craving, habit, sociability and increased need of energy or stimulation were described as motivators for smoking [21].

Risk of smoking in schizophrenia may depend on the type of antipsychotic. Typical antipsychotics such as haloperidol are associated with an increased risk of smoking. This may be explained by the increase of dopaminergic activity by nicotine, which compensates for the D_2_ blockade produced by antipsychotics [17,22]. However, the degree of dopamine blockade by typical antipsychotics varies widely.

Several studies have reported that clozapine reduces smoking in patients with schizophrenia [23-25]. When 12 patients treated with haloperidol were switched to clozapine, they reduced the number of cigarettes smoked [24]. Another study among outpatients found that there was a significant decrease in daily cigarette use after clozapine treatment, as compared to treatment with typical neuroleptics. The decrease was confined to heavy smokers [25]. Clozapine has also been found to decrease alcohol use among patients with schizophrenia [23]. The unique mechanism of action of clozapine may be responsible for the reduced risk of substance use in patients with schizophrenia. Clozapine binds more strongly to D_4_ dopamine receptors than to D_2_ receptors, and it is also more effective than other antipsychotics in treating cognitive deficits in schizophrenia [26]. It has also been hypothesized that clozapine may reduce craving.

Although there is evidence that antipsychotics influence rates of smoking in patients with schizophrenia, the evidence is based on small intervention studies. This study had two main objectives. One was to identify the prevalence of smoking among patients with schizophrenia in a country with low overall prevalence of smoking. The other objective was to identify the association between smoking and antipsychotic treatment.

## Methods

This cross-sectional study was carried out in the psychiatry outpatient clinics (including a specialized clozapine clinic) in a tertiary care hospital in Sri Lanka. Sri Lanka is a middle-income country with a low prevalence of smoking among the general population. Among males in the general population, prevalence of smoking was 29.9% in urban areas and 24.4% in rural areas [27,28]. Smoking is rare among females.

Sample size was calculated to detect 40% prevalence with a precision of 0.6. The study population was patients registered in the outpatient clinics of the unit, with a diagnosis of schizophrenia according to ICD-10 clinical criteria. Every third patient was selected using systematic sampling.

Smoking behaviours were assessed using self-reports. Wherever possible, this information was cross checked with accompanying family members. Current tobacco use was defined as smoking cigarettes or *beedi* (traditional Indian cigarettes) or use of smokeless tobacco (tobacco used in betel quid). Current tobacco use and current smoking were defined as use within the last 30 days. Severity of illness was assessed using BPRS. Fagerstrom Test for Nicotine Dependence assessed level of dependence. Readiness to Change Questionnaire assessed motivation to change smoking behaviour [29].

Written informed consent was obtained from all patients who participated in the study. Ethical clearance was obtained from the Ethics Review Committee of the National Hospital of Sri Lanka. Data analysis was carried out using SPSS-14 computer software. Chi-square tests assessed association between categorical variables. One-way ANOVA and independent *t* tests assessed difference between means.

## Results

### Description of the sample

The sample consisted of 306 patients. Mean age was 38.93 years (SD 10.98). There were 148 males (48.4%). Mean duration of illness was 12.63 years (SD 8.38). Current medication was risperidone 74 (24.2%), olanzapine 50 (16.3%), trifluoperazine 32 (10.5%), haloperidol 6 (1.9%), chlorpromazine 14 (4.6%), clozapine 136 (44.4%) and depot fluphenazine 41 (13.4%). Eight patients on either risperidone or olanzapine were also treated with an oral typical antipsychotic. Polypharmacy was commonest among patients treated with a depot antipsychotic with 8 (19.5%) also treated with risperidone or olanzapine and 10 (24.4%) treated with an oral typical antipsychotic. Eleven patients (8.08%) on clozapine were also prescribed another atypical antipsychotic, while concomitant trifluoperazine use was recorded in two (0.65%). Mood stabilizers were prescribed in 13 (4.25%). Patients who were on clozapine were refractory to treatment with at least two other antipsychotics.

### Tobacco use

Prevalence of current tobacco use (smoking and smokeless tobacco use) was 15.69% (95% CI 11.59 to 19.78). Prevalence of tobacco use among males was 30.41% (95% CI 22.91 to 37.90) and among females 1.90% (95% CI -0.25 to 4.05). Prevalence of current smoking among males was 20.27% (95% CI 13.72 to 26.82). None of the females smoked. Prevalence of smokeless tobacco use among males was 10.14% (95% CI 5.22 to 15.05) and among females 1.90% (95% CI -0.03 to 4.05). The mean number of cigarettes or beedis smoked daily was 8.81 (SD = 2.91).

When patients treated with clozapine were excluded from the analysis, prevalence of tobacco use increased to 41.6% among males and 3.2% among females and the prevalence of smoking among males was 29.9%.

Table 1 compares characteristics of tobacco users and non-users. Although mean duration of illness was longer in tobacco users, the difference was not significant (*t* = 0.75, *p* = 0.42). There was no significant difference in mean BPRS score between tobacco users 32.06 (SD 9.17) and non-users 32.12 (SD 9.02) (*t* = 0.38, *p* = 0.97). There was no significant difference in BMI between tobacco users 22.17 (SD 3.22) and non-users 23.76 (SD 4.11) (*t* = 2.53, *p* = 0.12).

**Table 1:** Comparison of tobacco users and non-users

	**Tobacco users**	**Non-users**	**Significance**
Age in years, mean (SD)	41.56 (10.58)	38.44 (11.01)	*t* = 1.82, *p* = 0.07
Gender males (%)	93.8	39.9	*χ*^2^ = 46.95, *p* = 0.001
Duration of illness, mean (SD)	13.54 (9.27)	12.47 (8.21)	*t* = 0.81, *p* = 0.42
BPRS, mean (SD)	31.84 (8.99)	32.09 (8.81)	*t* = 0.16, *p* = 0.88
Body mass index, mean (SD)	22.17 (3.22)	23.77 (4.11)	*t* = 2.53, *p* = 0.63

Dependence was assessed using the Fagerstrom Test for Nicotine Dependence. Among current smokers, 23 (76.6%) had low-moderate dependence and 7 (23.3%) high dependence. Among smokeless tobacco users, 13 (72.2%) had low-moderate dependence and 5 (27.7%) high dependence. There was no significant difference in the level of dependence among smokers and smokeless tobacco users (*χ*^2^ = 0.13, *df* = 2, *p* = 0.94). Among tobacco users, 43 (89.5%) were daily users. Among smokers, 14 (46.6%) smoked ≤5 cigarettes/day. All those who were highly dependent smoked ≥10 cigarettes/day.

Readiness to Change Questionnaire assessed the motivation to change. Of the tobacco users, 19 (41.3%) were in the pre-contemplation stage, 12 (26.1%) were in the contemplation stage and 15 (32.6%) in the action stage.

### Antipsychotics and tobacco use

Because none of the females smoked and prevalence of smokeless tobacco use was also low among females, we included only males in the analysis of the relationship between tobacco use and antipsychotics. Table 2 shows that prevalence of tobacco use was lowest in patients treated with clozapine 18.31 (95% CI 9.09 to 27.53) and highest in those treated with depot antipsychotics 47.83 (95% CI 25.74 to 69.91). Logistic regression analysis shows that among males, risk of tobacco use was significantly lower in patients treated with clozapine [OR 0.36 (95% CI 0.16 to 0.84)]. This is despite the fact that patients treated with clozapine had higher BPRS mean scores 34.57 (SD11.28) than those on atypical antipsychotics 30.46 (SD 5.63) or depot antipsychotics 29.30 (SD 4.96).

**Table 2:** Demographic and clinical variables according to current antipsychotic treatment

	**Atypical antipsychotics *n*** **= 103**	**Clozapine *n*** **= 136**	**Oral typical antipsychotics *n*** **= 29**	**Depot typical antipsychotics *n*** **= 38**
Age years (SD)	39.25 (11.47)	34.54	47.69	47.05
Gender males (%)	45.6	52.2	24.1	60.5
Illness duration years in males (SD)	11.75 (8.81)	12.17 (6.63)	18.42 (8.22)	19.83 (8.0)
BPRS mean in males (SD)	31.87 (6.07)	34.33 (11.67)	28.67 (7.87)	28.88 (4.57)
Prevalence of tobacco use among males (95% CI)	38.30 (23.87 to 52.72)	18.31 (9.09 to 27.53)	42.86 (-6.58 to 92.29)	47.83 (25.74 to 69.91)

There was significant difference in mean age (*F* = 25.16, *p* < 0.001), duration of illness (*F* = 10.85, *p* < 0.001) and mean BPRS scores (*F* = 4.20, *p* < 0.001) between different antipsychotic groups. Patients on oral and depot typical antipsychotics tended to be older and had had longer duration of illness than those on atypical antipsychotics.

## Discussion

We found that the prevalence of tobacco use among males treated with clozapine was half that of patients on other antipsychotics. This finding is of importance because the sample included a large number of patients on clozapine, whereas previous evidence is mostly based on intervention studies with small samples [23-25]. The highest prevalence of tobacco use among males was in the depot antipsychotic group.

The exact mechanism by which clozapine reduces smoking is not very clear. Clozapine may reduce smoking through its specific action of reducing cognitive deficits. Another possibility is that the improved therapeutic response to clozapine also reduces risk of smoking. A group of treatment refractory patients on haloperidol after treatment with therapeutic doses of clozapine showed lower BPRS scores and reduced the number of cigarettes smoked [24]. However, our findings do not support this because the patients on clozapine had higher mean BPRS scores than those on other antipsychotics.

There is also evidence from a few other studies that the type of antipsychotic influences the smoking rate. A study of 39 inpatients with schizophrenia spectrum disorder found that patients on typical antipsychotics had the highest prevalence and those on clozapine the lowest [25,30]. Typical antipsychotics are probably less effective than clozapine in correcting abnormal sensory processing and cognitive impairment associated with schizophrenia. Typical antipsychotics are more likely to cause extrapyramidal symptoms (EPS), and nicotine may reduce neuroleptic induced EPS [31,32].

Although many biological mechanisms have been proposed to explain the increased prevalence of smoking in schizophrenia, our study suggests that sociocultural factors are also important [8]. This study was conducted in a country where overall prevalence of smoking among the general population is not high [27,28,33]. The prevalence of smoking in our study was lower than that reported in most other countries. When patients treated with clozapine were excluded, prevalence of tobacco use among males was 41.6% and the prevalence of smoking was 29.9%. This is similar to the prevalence of smoking among the general population of 29.9% [27,28].

Social factors can explain the relatively low prevalence of smoking in our study. In low- and middle-income countries, affordability significantly influences smoking rates [34]. A study from India reported that lack of economic independence and restrictions imposed by the family appeared to be crucial factors that controlled the prevalence of smoking among patients with schizophrenia [34]. In Sri Lanka, patients with schizophrenia do not receive a disability allowance [35]. Most of the unemployed patients are dependent on their families. Therefore, they may not be able to afford to purchase cigarettes. A previous study of outpatients in the same unit treated with clozapine shows that 55.6% of the males were unemployed [36]. Another study of patients with schizophrenia attending follow-up clinics showed that 42.9% of males reported a monthly income of less than 150 US$ [35]. Low affordability is supported by the fact that 10.1% of males used smokeless tobacco in the form of betel quid, which is cheaper than cigarettes. This is much higher than the prevalence of 1.7% reported among the general population in urban areas [37]. Betel quid contains betel leaf (Piper betle), arecanut (Areca catechu) slaked lime (calcium hydroxide) and tobacco [38]. Influence of cultural factors is also apparent because females in our sample did not smoke at all which is similar to the pattern in the general population [39].

Also, in high-income countries, social factors influence prevalence of smoking among the mentally ill. In these countries, cigarettes are more affordable to people with mental illness and smoking is also engrained in the culture of many institutions that care or provide for the mentally ill [1]. Many inpatient psychiatry units do not enforce smoke-free policies, and smoking may facilitate social interactions.

Several limitations must be considered in interpreting the findings of this study. This study used self-reports of tobacco use and smoking as in most epidemiological studies. Previous research has shown that information obtained from patients and informants underestimates substance use when compared to laboratory analysis [40,41]. Only data from males were used in the analysis of association between tobacco use and antipsychotics.

## Conclusions

This study identified two factors which influence tobacco use in schizophrenia. We found that clozapine is associated with reduced risk of smoking. The study also found that social factors significantly affect smoking rates in patients with schizophrenia. In a society with low smoking rate, the patients may not have been smokers to start; however, we do not have evidence to support this. We did not find an association between smoking and severity of illness although other studies have found such an association.

Policy makers should explore the possibility of restricting availability of cigarettes and adopting smoke-free policies in mental health units.

More research is needed to understand the relationship between antipsychotics and smoking in patients with schizophrenia. If other studies are able to replicate the finding of lower risk of smoking with clozapine, it could lead to a recommendation to consider clozapine as a treatment option for smokers with schizophrenia.

## Competing interests

The authors declare that they have no competing interests.

## Authors’ contributions

HW contributed to the design of the project and data collection. VAdeS and RH contributed to the design of the project, supervision of data collection, analysis of data and writing of the paper. All authors have read the final draft and are in agreement with the content of the manuscript.
